# Multimodal Autonomous Locomotion of Liquid Crystal Elastomer Soft Robot

**DOI:** 10.1002/advs.202402358

**Published:** 2024-03-23

**Authors:** Xiaorui Zhou, Guancong Chen, Binjie Jin, Haijun Feng, Zike Chen, Mengqi Fang, Bo Yang, Rui Xiao, Tao Xie, Ning Zheng

**Affiliations:** ^1^ State Key Laboratory of Chemical Engineering College of Chemical and Biological Engineering Zhejiang University Hangzhou 310027 China; ^2^ State Key Laboratory of Fluid Power and Mechatronic Systems Key Laboratory of Soft Machines and Smart Devices of Zhejiang Province Department of Engineering Mechanics Zhejiang University Hangzhou 310027 China

**Keywords:** autonomous locomotion, liquid crystal elastomer, smart material, stimuli‐responsive polymers

## Abstract

Self‐oscillation phenomena observed in nature serve as extraordinary inspiration for designing synthetic autonomous moving systems. Converting self‐oscillation into designable self‐sustained locomotion can lead to a new generation of soft robots that require minimal/no external control. However, such locomotion is typically constrained to a single mode dictated by the constant surrounding environment. In this study, a liquid crystal elastomer (LCE) robot capable of achieving self‐sustained multimodal locomotion, with the specific motion mode being controlled via substrate adhesion or remote light stimulation is presented. Specifically, the LCE is mechanically trained to undergo repeated snapping actions to ensure its self‐sustained rolling motion in a constant gradient thermal field atop a hotplate. By further fine‐tuning the substrate adhesion, the LCE robot exhibits reversible transitions between rolling and jumping modes. In addition, the rolling motion can be manipulated in real time through light stimulation to perform other diverse motions including turning, decelerating, stopping, backing up, and steering around complex obstacles. The principle of introducing an on‐demand gate control offers a new venue for designing future autonomous soft robots.

## Graphical abstract

A liquid crystal elastomer soft robot capable of self‐sustained continuous movement, with a specific motion mode that can be gate‐controlled by either substrate adhesion or remote light. It is mechanically trained to undergo continuous snapping actions to ensure its self‐sustained locomotion. By on‐demand setting, the autonomous soft robot can perform sophisticated tasks, including rolling, jumping, making stops, decelerating, backing up, and even turning around obstacles.

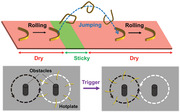

## 1 Introduction

Soft robots offer great potential to accomplish challenging tasks that conventional rigid robots cannot.^[^ ^1^, ^2^, ^3^, ^4^, ^5^ ^]^ Their softness and agility bear similarities to humans and other biological organisms. Naturally, their design principles are heavily influenced by biological species.^[^ ^6^, ^7^, ^8^, ^9^, ^10^ ^]^ The actuation of soft robots typically originates from soft materials that can actively respond to external stimuli.^[^ ^11^, ^12^, ^13^, ^14^, ^15^, ^16^ ^]^ As such, external controls are typically required, such as cooling‐heating^[^ ^17^ ^]^ and on‐off switching of light,^[^ ^18^, ^19^ ^]^ electrical field,^[^ ^20^, ^21^ ^]^ and magnetic field.^[^ ^22^, ^23^ ^]^ Multimodal motions can also be achieved by continuously switching these stimuli.^[^ ^24^, ^25^ ^]^ These external controls offer a means for active manipulation that is beneficial in many circumstances. They do, however, place severe restrictions on others due to the lack of device autonomy.

Self‐oscillation (e.g., heartbeats and sea waves) which is ubiquitous in nature, offers unusual inspiration for designing synthetic systems capable of autonomous movement.^[^ ^26^, ^27^, ^28^, ^29^ ^]^ It refers to a phenomenon of periodic motions (e.g., back and forth) under a constant condition. Because it does not require altering the external condition or control, such periodic motion is considered self‐sustained,^[^ ^30^ ^]^ in contrast to typical behaviors of shape‐shifting materials that require changing the external conditions (e.g., heating and cooling) for sustained movement. Mechanistically, self‐oscillation is an out‐of‐equilibrium process originating from a negative feedback loop internal to a system that allows periodic conversion between two kinetically stable states under a constant condition. This unique mechanism has attracted enormous attention for designing autonomous moving synthetic systems, which demonstrate distinct motions such as self‐walking, self‐rotation, or self‐rolling. Responsive hydrogels driven by the Belousov‐Zhabotinsky oscillating reaction can be designed to possess self‐walking capability.^[^ ^31^ ^]^ Under constant light illumination, light‐responsive liquid crystal elastomers (LCEs) can exhibit various self‐sustained actions (wave‐making and continuous rotation) depending on the geometric system designs.^[^ ^32^, ^33^, ^34^, ^35^, ^36^ ^]^ A more generally applicable mechanism relies on a gradient thermal field to trigger autonomous rolling,^[^ ^37^, ^38^, ^39^, ^40^ ^]^ where the self‐sustained movement arises from a negative feedback loop between the shape‐shifting of an LCE and the heating‐cooling cycle due to the constant temperature gradient.

The above self‐sustained movements offer enormous appeal in the design of autonomous soft robots. However, the types of autonomous motions are inherently determined by fixed external conditions (stimulation). As such, each fabricated robot is only capable of a single fixed (albeit repeated) motion. In other words, they lose active controllability and multi‐motion capability. An ideal solution is to combine autonomous motions with more classical on‐demand control methods. Achieving this goal is challenging, as it requires designing not only a feedback loop essential for autonomous movement but also a system that is trigger‐responsive for other motions, with each able to positively interfere with the other. In this work, we realize this goal by designing an LCE that is both thermal and photo‐responsive. The LCE is mechanically trained to undergo thermally driven continuous shape‐shifting for self‐sustained locomotion. More importantly, remotely accessible light triggering offers an on‐demand mechanism to alter the locomotion mode in an easily controllable manner, differing from the typical use of light for powering.^[^ ^35^, ^41^ ^]^ We note that light stimulation of LCE allows for achieving multi‐modal motions via spatio‐temporal control, but such a mechanism requires light continuous stimulation.^[^ ^25^, ^42^ ^]^ Therefore, the LCE can switch the locomotion mode in real‐time during its self‐sustained movement. In particular, we show that spatiotemporal light control allows the LCE robot to mimic the functions of a car, performing various sophisticated actions including turning, decelerating, stopping, backing up, and steering around obstacles. Additionally, we illustrate that by adjusting the adhesion between the LCE and the substrate, it is capable of reversibly transitioning from a rolling/running motion to jumping. These precisely controllable multi‐modal motions stand in sharp contrast to current autonomous soft robotic systems.

## 2 Results

### 2.1 Synthesis and Mechanical Training of LCE

**Figure 1:**
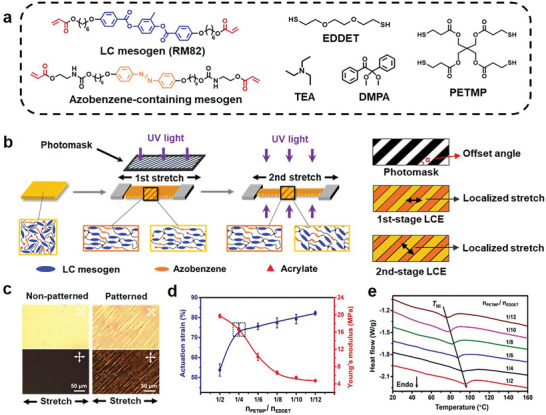
LCE synthesis and mechanical training. a) Precursors of the LCEs. b) Schematic illustration of the mechanical training process. c) Polarized optical micrographs of non‐patterned and patterned LCE films. d) The dependence of actuation strain and Young's modulus on the crosslinker content. e) The DSC curves of LCEs with different crosslinker content. All error bars in this figure correspond to SD (n = 3).

We start by fabricating a polydomain LCE film using four monomer precursors (**Figure** **1**a): 1) a liquid crystal mesogen (RM82); 2) a photo‐responsive liquid crystal mesogen (azobenzene‐containing diacrylate); 3) a dithiol (EDDET); 4) a tetra‐thiol cross‐linker (PETMP). Triethylamine (TEA) and a photo‐initiator (DMPA) are also included as catalysts/initiators. The molar ratio of the total thiol and acrylate groups is fixed at 0.9. While the photo‐responsive liquid crystal mesogen is necessary for light sensitivity, it could compromise the thermal response. Hence, the photo‐responsive mesogen is kept at 5% (Figure S1, Supporting Information) of the thermal responsive liquid crystal mesogen (RM82) to ensure the dominance of the latter. With such a mixture, thermal curing triggers the thiol‐acrylate Michael click reaction, yielding a lightly crosslinked LCE network. The network can be further cured under UV light through the radical polymerization of the remaining acrylate moieties to yield a densely crosslinked network.

The mechanical training of the LCE is conducted at room temperature. The lightly crosslinked polydomain LCE film before UV curing is subjected to a two‐stage process (Figure 1b). In Stage 1, the film is uniaxially stretched to a first strain (Ɛ_1_) and cured under masked UV light. The orange and yellow colors in Figure 1b denote the masked and non‐masked regions, respectively. In Stage 2, the sample is further stretched to a second strain (Ɛ_2_, compared to the initial sample length) and UV‐cured without photo masking. Here, the curing time for both stages is kept identical at 5 min given the optimal actuation of the LCE produced (Figure S2, Supporting Information). During both stages, UV curing allows for locking the mechanically induced mesogen alignment. After Stage 1, the orange region becomes more rigid due to the UV curing, causing the strain applied in Stage 2 to be predominantly located in the yellow region. This allows the orange and yellow regions to lock the alignment from Ɛ_1_ and Ɛ_2_, respectively. As Ɛ_2_ >Ɛ_1_, the degree of alignment in the yellow region is higher than in the orange region. The mechanical properties of the masked and non‐masked regions after Stage 1 and Stage 2 are shown in Figures S3 and S4 (Supporting Information), respectively. After Stage 1 curing, the non‐masked region exhibits a notably higher modulus compared to the masked region due to the higher degree of polymerization. However, after Stage 2 curing, this difference in modulus becomes insignificant.

In the above design, there are many variables one can explore, including the ratio between the two regions and their spatio‐distribution, both of which are determined by the mask. For simplicity, we focus on rectangular strip patterns with the total masked area kept equal to the non‐masked area. Additionally, the width of the strip is maintained equally in different regions. Within this design, the only two variables are the offset angle and width of the rectangular strip. The offset angle is particularly important here. When it is neither 0 nor 90 °, even if the global stretching direction remains consistent during the entire mechanical training process, the localized mesogens alignment in the yellow region becomes offset from the global stretching direction. In contrast, the alignment direction in the orange region is solely determined by the first stretching and therefore remains identical to the global stretching. Overall, upon the completion of the two‐stage training, an LCE with spatially defined alignment degree and direction is obtained.

We focus on an LCE sample that is photo‐mechanically trained with Ɛ_1_ of 80%, an offset angle of 45 °, and a strip width of 10 µm. Under this condition, Ɛ_2_ has a large impact on the actuation (Figure S5, Supporting Information). We pick Ɛ_2_ of 116% for future investigation, considering the optimal actuation performance. The thickness of the LCE sample is reduced to 0.36 mm after the mechanical training. A non‐patterned reference sample is prepared identically, except that no mask is employed. The polarizing optical microscope (POM) images (Figure 1c) show that the non‐patterned reference is aligned in the direction of stretching. In contrast, the two regions in the patterned sample are also aligned, but their alignment directions are offset by nearly 45 °. The samples cured under different pattern widths show similar differentiation of alignment (Figure S6, Supporting Information).

For the dual‐cured network, we define the crosslinker content as the molar ratio between the tetra‐thiol crosslinker and the dithiol (n_PETMP_/n_EDDET_). Adjusting crosslinking allows for tuning the LCE networks. Figure 1d illustrates that, as the crosslinking decreases, the actuation strain of the LCE increases, but Young's modulus declines. The crosslinker content also impacts the temperature at which the transition occurs from the nematic phase to the isotropic phase (*T_NI_*), with a higher crosslinker resulting in a higher *T_NI_* within the range of 80 °C to 97 °C (Figure 1e). For our intended purposes, the LCE should possess sufficient actuation capability, yet its modulus should be high enough for self‐supporting. Therefore, we select a balanced value of crosslinker content (n_PETMP_/n_EDDET_ = 1/4, *T_NI_*: 93 °C) for subsequent experiments.

### 2.2 Self‐sustained Rolling Under Thermal Field

**Figure 2:**
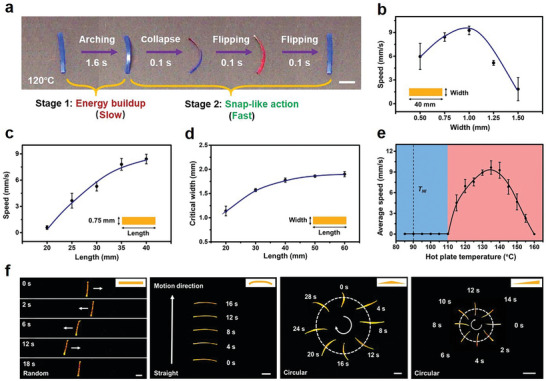
Self‐sustained continuous motions. a) The real‐time images of the LCE in one movement cycle. b) The dependence of moving speed on the sample width. c) The dependence of moving speed on the sample length. d) The impact of sample length on the critical width. e) The influence of hot plate surface temperature on the moving speed. f) The motion behaviors of LCEs with different geometries. All the scale bars are 1 cm. All error bars in this figure correspond to SD (n = 3).

When the mechanically trained LCE is placed on a hotplate with the surface temperature set at 120 °C, it exhibits self‐sustained continuous movement (Movie S1, Supporting Information), although the movement direction is random. **Figure** **2**a captures the detailed actions within a single movement cycle, with the two sides of the LCE colored in red and blue for distinction. Starting from a flat rectangular film (length: 40 mm, width: 1.5 mm), the LCE gradually arches up over 1.6 s. Afterwards, it collapses suddenly followed by two consecutive flipping actions, all occurring very rapidly (within 0.1 s). The net result is that the LCE moves, but its geometry returns to its initial flat state with the blue side facing up. The cycle of actions repeats continuously without any external interference, enabling continuous autonomous moving.

We posit that the self‐sustained movement stems from the thermal field. Although the hotplate surface is set at a fixed temperature, there naturally exists a temperature gradient in the air surrounding the hotplate. Upon initial contact with the hotplate, the LCE is heated above its *T_NI_* and contracts. The bottom side experiences heating and contraction before the upper side, causing upward arching. At a localized level, the yellow and orange regions contract in different directions due to their distinct alignment directions. These different contraction directions induce internal stress within the LCE, leading to additional arching. This is corroborated by finite element analysis (Figure S7, Supporting Information), which demonstrates good consistency with our experimental observations. This upward arching action is akin to the behavior observed in hydrogels^[^ ^43^, ^44^ ^]^ with similar patterned stress fields. Simultaneously, the LCE naturally cools as it moves away from the hotplate surface. Once the up‐arching reaches a critical point, the LCE loses balance and collapses in a snapping‐like manner. Driven by the inertia, the LCE flips twice to return to its initial state, resulting in movement. This cycle of actions repeats, enabling continuous motion. The patterning plays a crucial role in this process. In the absence of patterning, a uniformly aligned LCE would exhibit only minimal initial up‐arching without any self‐sustained movement (Figure S8, Supporting Information). Regarding the pattern, with a fixed Ɛ_1_ of 80%, and Ɛ_2_ of 116%, we investigated the impact of the offset angle and pattern strip width, with the results presented and discussed in Supplementary Materials (Figures S9 and S10, Supporting Information). Subsequently, the optimal set of parameters (Ɛ_1_ of 80%, Ɛ_2_ of 116%, offset angle of 45 °, and pattern strip width of 10 µm) is utilized. The produced LCE demonstrates stable actuation performance upon direct heating and cooling (Figure S11, Supporting Information).

We then proceed to optimize the motion speed by altering the rectangular geometry of the sample. While the motion direction tends to be somewhat random, there is a section of the continuous motion process where the LCE moves consistently in one direction. This section is captured in video recordings and utilized to determine the average moving speed. It is essential to note that the overall motion speed is primarily influenced by the initial slow up‐arching step. To assess the impact of sample width on motion speed, we maintain the sample length at 40 mm and evaluate a series of LCE samples with varying widths. The results (Figure 2b) indicate an initial increase in speed with increasing width, peaking at nearly 9.3 mm s^−1^ with a sample width of 1 mm. Beyond this critical width, however, the speed begins to decline. This trend arises from the balance of two width‐related factors. First, due to the fixed sample thickness (0.36 mm), the up‐arching process is easier for a wider sample from a bending energy standpoint, which favors faster speed. Conversely, wider samples are more stable and resistant to collapse, which impedes motion. The overall effect is such that there is an optimal sample width for the maximum speed. Moreover, when the width is fixed, the sample length significantly affects the speed (Figure 2c). Notably, the speed increases almost linearly with the length. This is because, within the investigated range from 20 to 40 mm, a longer sample can lose balance and reach the collapse point earlier, leading to faster overall speed. Additionally, we observed a critical width threshold above which the LCE only up‐arches without flipping continuously for motion. This critical width increases with the sample length (Figure 2d).

The speed is also influenced by the surface temperature of the hotplate (Figure 2e). Below 110 °C, no continuous motion is observed. As the temperature surpasses this threshold, continuous movement initiates, with the speed increasing proportionally to the temperature rise. It reaches a maximum speed at 135 °C above which the motion starts to become slower until no motion at 160 °C. These experimental results are consistent with the mechanism proposed earlier. First, the temperature must exceed the *T_NI_* of the LCE to occur the motion. Second, the temperature of the LCE's upper surface should be sufficiently below the *T_NI_* to facilitate cooling‐enhanced upward arching. This condition is not met when the hotplate temperature is excessively high. It is noteworthy that the LCE only begins to decompose at ≈300 °C (Figure S12, Supporting Information), ensuring its thermal stability within the temperature range of 110−160 °C. In addition, the actuation strain undergoes only a slight degradation (from 75% to 72%) after aging for 4 h at 130 °C.

The above continuous motion progresses with each movement occurring in a random direction (Figure 2f). In this case, the rolling direction is determined by the position of the sample's center of gravity. For a symmetrical rectangular sample, uncontrollable collapsing directions are observed since its center of gravity lies in the middle, resulting in random back‐and‐forth motion. However, any slight deviation from geometric symmetry can introduce a controllable motion direction. Indeed, when a flat LCE film is cut into a slightly curved shape (an in‐plane arch), it begins to move continuously and consistently in the direction of the arch (Figure 2f; Movie S2, Supporting Information). Additionally, an LCE film shaped like a triangular exhibits circular motion (Figure 2f; Movie S2, Supporting Information) due to the geometric bias, completing a full circle in 32 s. Altering the shape of the triangle enables the LCE to execute a much tighter circular motion in 16 s.

### 2.3 Autonomous Jumping Motions and Multi‐motion Combinations

**Figure 3:**
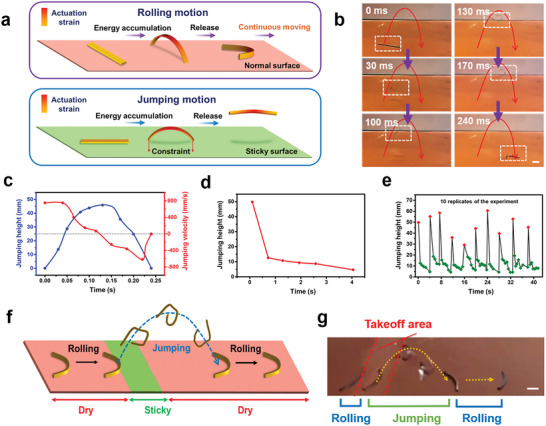
Autonomous jumping motions. a) Schematic illustration of the mechanism of rolling and jumping. b) Photographs of the LCE's time‐dependent jumping behavior. c) Jumping height and velocity of the LCE. d). Consecutive jumping within a single cycle. e) Ten replicates of consecutive jumping cycles (the red points correspond to the initial jump for each cycle and the green points represent the consecutive jumps within each cycle). f) Schematic illustration of multimodal autonomous locomotion. g) The combined autonomous rolling and jumping motions. All the scale bars are 1 cm.

Ideally, a robot should not only possess autonomous mobility but also the capability to perform different motions on demand. Among many possible motions, jumping is considered as one of the most demanding motions, requiring high energy power. While examples of jumping robots are known in the literature,^[^ ^45^ ^]^ they typically necessitate changing stimulation conditions to induce jumping actions (e.g., heating or light).^[^ ^46^, ^47^, ^48^, ^49^, ^50^, ^51^, ^52^ ^]^ Thus, autonomous jumping robots that can execute the motion under constant conditions are rare. In particular, Crosby's group reported a solvent evaporation induced autonomous jumper, but it is limited to this single motion.^[^ ^53^ ^]^ Currently, no example exists of an autonomous robot capable of multiple motions. The continuous rolling, combined with energy buildup and snap‐like flipping (**Figure** **3**a), prompts speculation regarding whether manipulating the energy accumulation during the buildup stage could potentially yield different motions such as jumping. As illustrated in Figure 3a, the motion can be facilitated by incorporating a suitable constraint before snapping. Implementing this constraint poses a pivotal challenge. We envision regulating the constraint by tuning the adhesion between the hotplate and the LCE, achieved by placing LCE on a stickier surface. This would allow for more energy accumulation before the up‐arching motion. If the energy accumulation is sufficiently high, its sudden release would lead to a rapid up‐arching that could propel the LCE soft robot toward jumping.

To demonstrate feasibility, we fabricated an axisymmetric strip structure with the two sides colored in black for distinction and placed it on a hotplate surface coated with viscous silicone oil. Gratifyingly, we found that the LCE is indeed capable of jumping in a parabolic fashion (Figure 3b), with individual actions captured more clearly in a slow motion video (Movie S3, Supporting Information). The jumping action is complete within 0.24 s, with maximum take‐off velocity and jumping height reaching nearly 0.8 m s^−1^ and 45 mm (Figure 3c). We verified our theoretical model of jumping process by finite element analysis, showing good consistency with the mechanism we proposed (Figure S13, Supporting Information). According to the mechanism, the LCE is theoretically capable of repeated jumping since it is capable of absorbing energy from the hotplate. Indeed, the LCE robot undergoes multiple consecutive jumps, although the jumping height decreases cycle after cycle (Figure 3d), ultimately losing its jumping capability after six jumps. We believe this is because the LCE only remains in the air for a very short time during jumping (≈0.2 s), hindering it from fully recovering to its original state through natural cooling. This results in a decrease in the energy that it can gain when it is dropped onto the hotplate. In principle, the consecutive jumping performance can be improved by prolonging its in‐air time, which can be realized with higher initial jumping with an LCE capable of higher actuation stress. Following this strategy, it is even possible to achieve endless jumping. This is an interesting direction we will pursue in the future. For the current system, after completing a full autonomous cycle, the LCE is taken away from the hotplate and fully cooled under ambient conditions. The LCE would regain the jumping capability. Figure 3e shows ten replicates of the jump cycles using the same sample, indicating the LCE is capable of repeated jumps in each cycle despite some variations in the jumping performance.

The next question is: can the rolling and jumping motions be combined in an autonomous cycle? To demonstrate this feasibility, we fabricated a planar arched LCE (40 mm × 1 mm × 0.36 mm) and put it on a hotplate with certain areas coated with silicone oil. The area named the “takeoff line” is coated with silicone oil. When the LCE robot rolls and reaches the takeoff line, it jumps forward like a long jumper. Afterward, the LCE continues to roll like a runner (Figure 3f,g; Movie S4, Supporting Information). Besides the “running‐jumping‐running” locomotion, the LCE is also capable of “jumping‐jumping‐running” when it is placed on the takeoff line directly (Movie S4, Supporting Information).

### 2.4 On Demand Light Control of Multimodal Autonomous Motions

**Figure 4:**
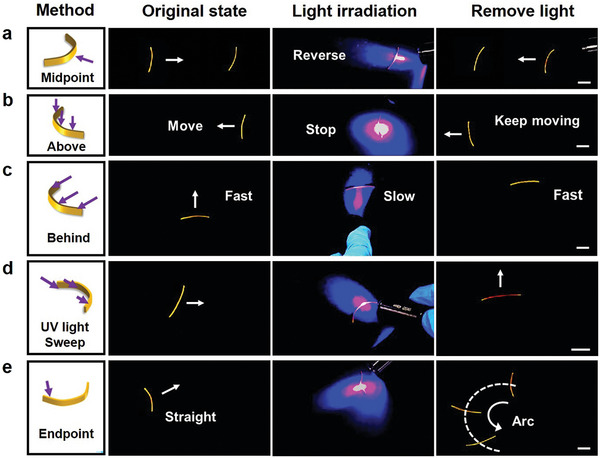
Light‐gated autonomous motions. a) Reversing the movement direction via irradiating the midpoint of the LCE. b) Stopping the movement via irradiating the top of the LCE. c) Deceleration via irradiating the backside of the LCE. d) Making a turn via light sweeping repeatedly from one end to the other. e) Changing from straight‐line moving to circular motion by irradiation of the sample endpoint for 5 s. All the scale bars are 1 cm.

Another mechanism to extend our self‐sustained LCE robot from a single motion to multi‐modal motions is to utilize the photo‐response of the LCE arising from the azobenzene moieties. We focus on a planar arched LCE sample (40 mm × 1 mm × 0.36 mm) to demonstrate its capability of self‐sustained multi‐modal motions gated by light control. As the LCE moves in its natural straight line, light is irradiated on different sections of the LCE. Upon irradiation on the mid‐point of the arched side (**Figure** **4**a) briefly (≈1 s), the LCE starts to move in the opposite direction continuously after the light is removed (Movie S5, Supporting Information), mimicking the backing up of a car. When light is irradiated fully from above, a moving LCE “car” would stop. It starts to move again as soon as the light is off (Figure 4b; Movie S5, Supporting Information). Likewise, when the light is irradiated from behind the arch (Figure 4c) or swept across the sample along the long axis (Figure 4d), the LCE shows two distinctively different actions (deceleration and turning, respectively). The above light irradiation is brief, and the “car” returns to the original locomotion rapidly after the light removal. In contrast, if the light exposure on one end is continuous for 4−5 s (Figure 4e; Movie S5, Supporting Information), the LCE exhibits a hysteresis, namely, it keeps a circular motion for roughly 15 s even after the light is off. After that, the “car” returns to its original straight movement. The sophisticated motion requires only simple and brief light stimulation, like a moving car receiving commands from the remote‐controlled operator.

The above multi‐modal motions originate from the localized LCE contraction based on *trans*‐to‐*cis* isomerization of the azobenzene unit.^[^ ^54^, ^55^ ^]^ This process effectively “disrupts” the natural thermal response of the LCE in a highly controllable manner. It should be noted that under such a short light exposure time (within 5 s), the photothermal effect can be ignored, as even prolonged light exposure for 60 s yields only a slight temperature increase of ≈2−3 °C (Figure S14, Supporting Information). When the light is turned off, the *trans*‐form reverts to the *cis*‐form rapidly at the elevated temperature, and the LCE returns to its original motion. However, when the extent of light‐induced isomerization is sufficiently high, the reverse process (*cis*‐to‐*trans* isomerization) does not occur instantaneously, resulting in hysteresis in the LCE's behavior.

**Figure 5:**
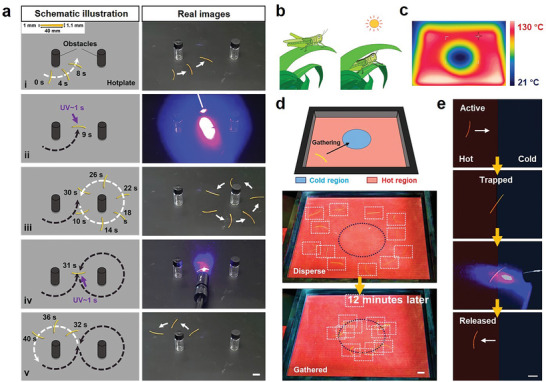
On‐demand light control of autonomous multimodal complexed motions. a) Moving around two obstacles. (Sample dimensions are illustrated in the inset image and the hot surface temperature is 130 °C). b) Thermotaxis of an insect. c) Infrared images of the thermal field showing the temperature distribution. d) Thermotaxis of our LCE samples. e) An LCE sample trapped at the hot‐cold boundary is released by light. All the scale bars are 1 cm.

The light‐gated autonomous motions of our LCE soft robot enable the execution of complex functions that pose challenges for known self‐sustained systems. In **Figure** **5**a, an LCE is depicted, cut into a trapezoidal shape with geometric asymmetry that naturally induces circular movement on a hotplate. This leads the robot to circumnavigate the first obstacle in a circular motion (step i). Upon brief light exposure at its front end, the robot turns around and enters the next circular orbit to navigate around a second obstacle (step ii). After repeating similar steps (iii and iv), the LCE robot performs a series of complex motions overall (Movie S6, Supporting Information) with its trajectory geometrically resembling the number “8”. Importantly, the light‐gated autonomous motions are not restricted to an air environment but can be extended to a water environment. Even when the hotplate is submerged in water (20 mm depth), our soft robot still exhibits self‐sustained continuous movement. Although its moving speed is reduced, possibly due to the higher drag force of water and/or differences in temperature gradient, it does not hinder the effectiveness of remote light triggering (Movie S7, Supporting Information).

Despite the versatility of these motions, soft robots like ours may face limitations in tasks involving high loads, unlike most conventional rigid robots. However, their compactness, simplicity, and flexibility offer unique benefits. In nature, there are numerous examples of insects that move relatively slowly and cannot carry substantial loads, resembling the characteristics of our soft robots. To emphasize this concept, we demonstrate the thermotaxis common in insects (Figure 5b) using our LCE robot, referring to their tendency to stay in a comfortable temperature region for survival. A hotplate with a non‐heated circular center (Figure 5c) is utilized to showcase the thermotaxis of our LCE as illustrated in Figure 5d. A series of LCE strips in random geometries are placed on the hotplate. These LCE strips move autonomously in a random fashion, yet most of them eventually become trapped (and stop) at the cold boundary (Figure 5d; Movie S8, Supporting Information). For the trapped LCE, it can be released with light, allowing it to move away from the center cold trap (Figure 5e; Movie S9, Supporting Information). This behavior mirrors insects swiftly escaping in response to external disturbances.

## 3 Discussion

In summary, our LCE robots are manufactured through a simple process involving stretching, masked UV curing, and cutting. They showcase self‐sustained and continuous rolling motion capabilities, due to the inherent feedback loop between a gradient thermal field and their shape‐shifting, with a snapping step offering the essential energy accumulation. When the energy accumulation reaches a sufficient level, the rolling can transition into jumping reversibly. In addition to thermally driven autonomous locomotion, the orthogonal photo‐response of our LCEs offers a unique gate control. Consequently, they can perform a diverse set of sophisticated motions/functions with real‐time light control. Our approach of combining self‐sustained motions with an easily accessible on‐demand control represents an attractive direction in designing future soft robots. For instance, it can be further integrated with lightweight flexible electronics with more sophisticated functions for exploration under harsh environments, such as hot roads and deserts.^[^ ^40^ ^]^ On the downside, our LCE requires high temperatures (above 110 °C) to operate, its actuation is limited by heat transfer, and switching the locomotion mode with light is performed manually. If desired, it is also feasible to lower the temperature requirement by tuning down the *T_NI_* of the LCE.^[^ ^56^ ^]^ These shortcomings pose challenges for it to work in any arbitrary environment. Further optimization should focus on reducing the actuation temperature and improving the heat‐transfer efficiency for faster moving speed. The hysteresis from the kinetics of *trans*‐to‐*cis* isomerization can be purposely enhanced such that the light‐interrupted locomotion can be sustained for a much longer time to extend the versatility of the robots.

## 4 Experimental Section

### Materials

The liquid crystal mesogen 1,4‐bis‐[4‐(6‐acryloyloxy‐hexyloxy) benzoyloxy]−2‐methylbenzene (RM82), was purchased from Beijing Bayi Space LCD Technology Co., Ltd. The chain extender 2,2′‐(ethylenedioxy) diethanethiol (EDDET) and phenol were obtained from Sigma‐Aldrich. The crosslinker pentaerythritol tetra(3‐mercaptopropionate) (PETMP) was procured from Rhawn. 2,2‐dimethoxy‐2‐phenylacetophenone (DMPA), 2‐isocyanatoethyl acrylate, and 4‐aminophenol and triethylamine (TEA) were purchased from TCI. The 6‐chloro‐1‐hexanol, potassium iodide, and sulfamic acid were purchased from Macklin. All the chemicals were used as received.

### Synthesis of the 4,4′‐Dihydroxyazobenzene

A 2 g of 4‐aminophenol was mixed with 8.4 mL of HCl (4 mL of 38% HCl were diluted to 8.4 mL) in a 25 mL round bottom flask for 1 h. The mixture was cooled to 0 °C in an ice bath. After 20 min, 10 mL of 2 m NaNO_2_/water solution was added dropwise. The reaction temperature was kept at 0 °C for 2 h and allowed to return to room temperature for 8 h. After the reaction, sulfamic acid was added to the mixture to quench the excess NaNO_2_. The solution was slowly added into another round bottom flask containing 1.72 g of phenol dissolved in a 2 mm NaOH/water solution. The reaction is allowed to proceed at room temperature for 12 h. Sulfamic acid was then added until precipitation occurred. The crude product was obtained after filtration and purified by dissolution in ethanol followed by precipitation in water. The product of 4,4′‐dihydroxyazobenzene was confirmed by ^1^H NMR analysis in DMSO‐d6 shown in Figure S15 (Supporting Information).

### Synthesis of 4,4′‐Bis(6‐Hydroxyhexyloxy) Azobenzene

A 3.08 g of 6‐chloro‐1‐hexanol, 1.61 g of 4,4′‐dihydroxyazobenzene, and 10.35 g of K_2_CO_3_ were mixed with 50 mL of DMF in a 150 mL three‐neck round‐bottom flask. After adding 0.01 g potassium iodide as the catalyst, the mixture was refluxed for 24 h under nitrogen. After cooling to room temperature, the reaction mixture was poured into 150 mL of distilled water and then filtered to obtain the crude product. The crude product was neutralized with HCl, and then filtered and washed with 150 mL of distilled water for 3 times. After evaporating water with oven heating, the crude product was recrystallized in chloroform and then dried at 70 °C under vacuum for 24 h, yielding the product of 4,4′‐bis(6‐hydroxyhexyloxy) azobenzene. Its structure was confirmed by ^1^H NMR analysis in DMSO‐d6 shown in Figure S16 (Supporting Information).

### Synthesis of the Azobenzene Containing Acrylate

A 3.38 g of 2‐isocyanatoethyl acrylate and 4.14 g of 4,4′‐bis(6‐hydroxyhexyloxy) azobenzene were mixed with 25 mL of DMF. The mixture was stirred and reacted at room temperature for 24 h. Methanol was added to the solution until precipitation occurred. The crude product was obtained after filtration, followed by recrystallization in chloroform for 3 times and drying at 70 °C under vacuum for 24 h. The obtained product was analyzed using ^1^H NMR in CDCl_3_ shown in Figure S17 (Supporting Information). Its UV‐vis spectrum of the azobenzene containing acrylate (100 ppm in DMF) illustrates peak absorbance at 365 nm shown in Figure S18 (Supporting Information).

### Synthesis of LCE Samples

A series of LCE samples with different molar ratios between the tetrathiol crosslinker and the dithiol (n_PETMP_/n_EDDET_) were synthesized using the same procedure except the n_PETMP_/n_EDDET_ being different. For the LCE with n_PETMP_/n_EDDET_ of 0.25, the synthetic process was described below. RM82 (1 g), azobenzene containing acrylate (0.05 g), EDDET (0.1893 g), PETMP (0.1269 g), and DMPA (0.07 g) were dissolved in DMF (1 g) at 80 °C. Next, 0.05 g of TEA was added to the solution. After fully mixed and degassed, the solution was poured in between two glass slides separated by a silicone rubber (thickness: 0.6 mm) and placed in an 80 °C oven overnight. After the reaction, the sample was dried in a vacuum oven at 70 °C for 12 h to fully remove DMF and TEA.

### Photo‐Mechanical Training of LCE

A polydomain LCE sample obtained in the previous step was placed on a tensile fixture with 80% pre‐stretch strain. The pre‐stretched LCE was covered with a photomask and exposed to 365 nm UV irradiation for 5 min. Next, the sample was placed on the tensile fixture again with an additional 20% pre‐stretch strain and irradiated with unmasked UV irradiation for 5 min on each side. Finally, the LCE was cut into the desired shape(s).

### Finite Element Analysis

FEA simulation was performed by using the commercial software Abaqus/Explicit to simulate the instability behavior of the LCE samples. The LCE model is split into two different regions according to oblique stripes photomask. The following elastic moduli (E) and Poisson's ratio (n) values were used for the simulation of LCE samples in their different areas (patterned region and non‐patterned region): E(Patterned) = 16 MPa, n (Patterned) = 0.3; E (Non‐patterned) = 13 MPa, n (Non‐patterned) = 0.3, with the moduli determined from the stress‐strain curves measured in experiments. The process of upward arching under thermal stimulus has been simulated. To simplify the model, it was assumed that the actuation strain of the two regions was the same and only the direction of deformation was different. The coupled temperature‐displacement linear (C3D8RHT) elements were used to mesh the structure. The boundary conditions were defined as an upper surface temperature of 25 °C and a lower surface temperature of 120 °C, and body heat flux Q_V_ = 4 was applied to the overall structure. The snap‐conducted jumping process was simulated in an explicit dynamic analysis process. The model was stretched to generate an arching displacement that mimics the morphing of LCE soft robots placed on the hotplate. Meanwhile, a downward constraint of both ends was utilized as the contact force between the LCE robots and the hotplate surface. This was to approximately simulated the behavior and interaction in a dynamic process under a constraint control in a simplified manner. In addition, gravitational force was also considered during the simulation.

### Characterization

The polarized optical images were collected using a polarized optical microscope (ECLIPSE E600W POL) at room temperature. The Young's modulus was measured using a Zwick/Roell tensile machine at a stretching speed of 10 mm min^−1^ at room temperature. The differential scanning calorimetry (DSC) analyses were conducted using a TA Q200 machine at a heating rate of 10 °C min^−1^. The absorption wavelength of azobenzene containing acrylate was measured by UV‐vis spectrophotometry (UV‐3150). ^1^H‐NMR spectra were collected using a Bruker Avance III machine (500 M). Thermogravimetric analysis (TGA, TA Q500) experiment was used to determine the decomposition temperatures of liquid crystal elastomer (LCE) samples under nitrogen protection at a heating rate of 10 °C min^−1^.

## Conflict of Interest

The authors declare no conflict of interest.

## Supporting information

Supporting Information

Supplemental Movie 1

Supplemental Movie 2

Supplemental Movie 3

Supplemental Movie 4

Supplemental Movie 5

Supplemental Movie 6

Supplemental Movie 7

Supplemental Movie 8

Supplemental Movie 9

## Data Availability

The data that support the findings of this study are available from the corresponding author upon reasonable request.
