# Supplementary material for: Multimodal Autonomous Locomotion of Liquid Crystal Elastomer Soft Robot
Source: Adv Sci (Weinh). 2024 Mar 23;11(23):2402358. doi: 10.1002/advs.202402358 (PMC11187929; doi:10.1002/advs.202402358)
Supplement: Supplementary file 1 — Supporting Information [file ADVS-11-2402358-s007.pdf]

ADVANCED SCIENCE
Open Access

Supporting Information

for *Adv. Sci.*, DOI 10.1002/advs.202402358

Multimodal Autonomous Locomotion of Liquid Crystal Elastomer Soft Robot

*Xiaorui Zhou, Guancong Chen, Binjie Jin, Haijun Feng, Zike Chen, Mengqi Fang, Bo Yang, Rui Xiao, Tao Xie and Ning Zheng**

Copyright WILEY-VCH Verlag GmbH & Co. KGaA, 69469 Weinheim, Germany, 2016.

Supporting Information

Multimodal Autonomous Locomotion of Liquid Crystal Elastomer Soft Robot

*Xiaorui Zhou[a]†, Guancong Chen[a]†, Binjie Jin[a], Haijun Feng[a], Zike Chen[b], Mengqi Fang[a], Bo Yang[a], Rui Xiao[b], Tao Xie[a], Ning Zheng[a]**

[a]State Key Laboratory of Chemical Engineering, College of Chemical and Biological Engineering, Zhejiang University, Hangzhou 310027, China.
[b]State Key Laboratory of Fluid Power and Mechatronic Systems, Key Laboratory of Soft Machines and Smart Devices of Zhejiang Province, Department of Engineering Mechanics, Zhejiang University, Hangzhou 310027, China.

†These authors contributed equally to this work.

E-mail: zhengning@zju.edu.cn

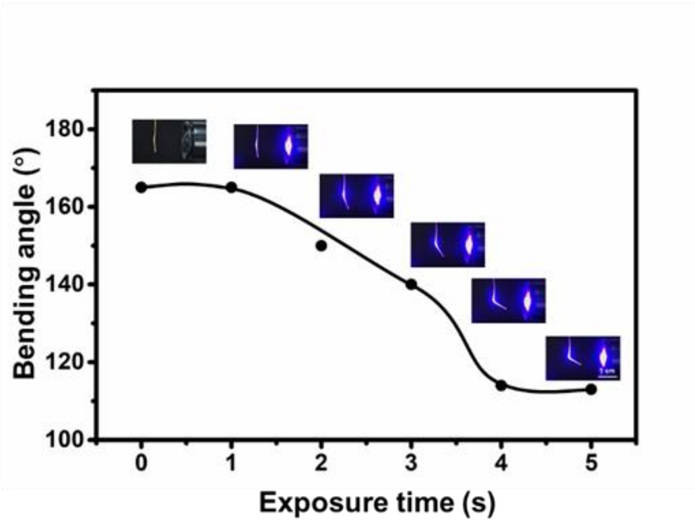

Figure S1. The UV light responsiveness of a 5% photo-responsive mesogen containing LCE (20mm×1mm×0.36mm) under constant irradiation (scale bar: 1 cm).

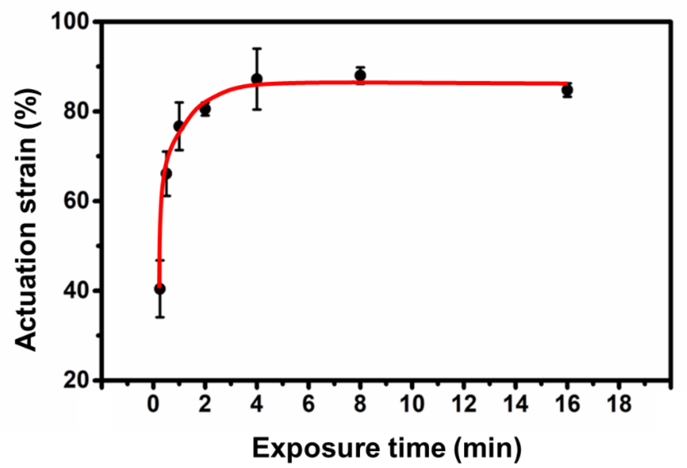

Figure S2. The dependence of actuation strain on the UV curing time. The sample is subjected to UV curing under a pre-stretch strain of 120%.

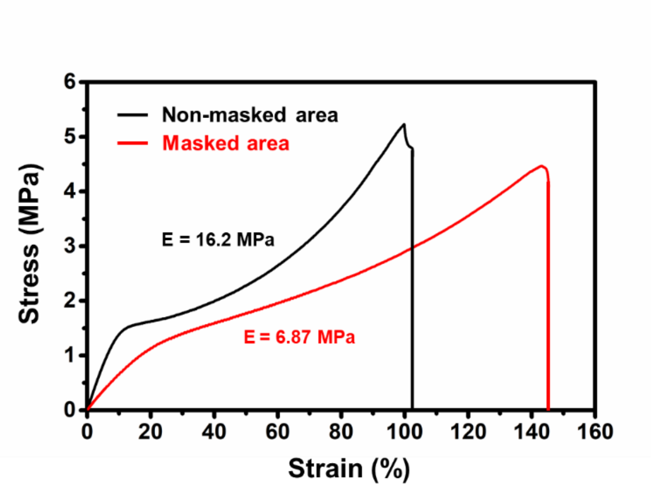

Figure S3. The mechanical properties of masked and non-masked regions after Stage 1 curing.

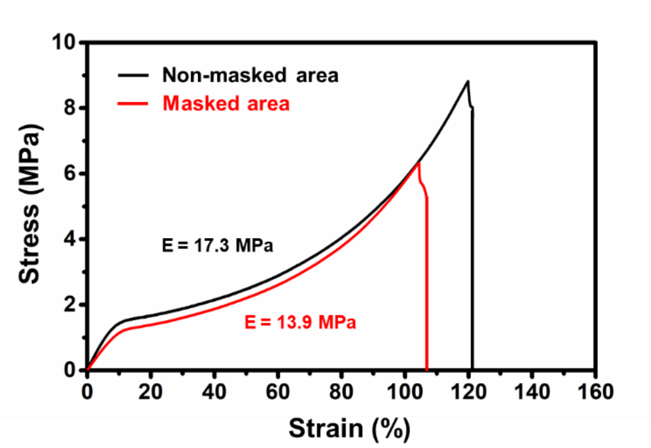

Figure S4. The mechanical properties of masked and non-masked regions after Stage 2 curing.

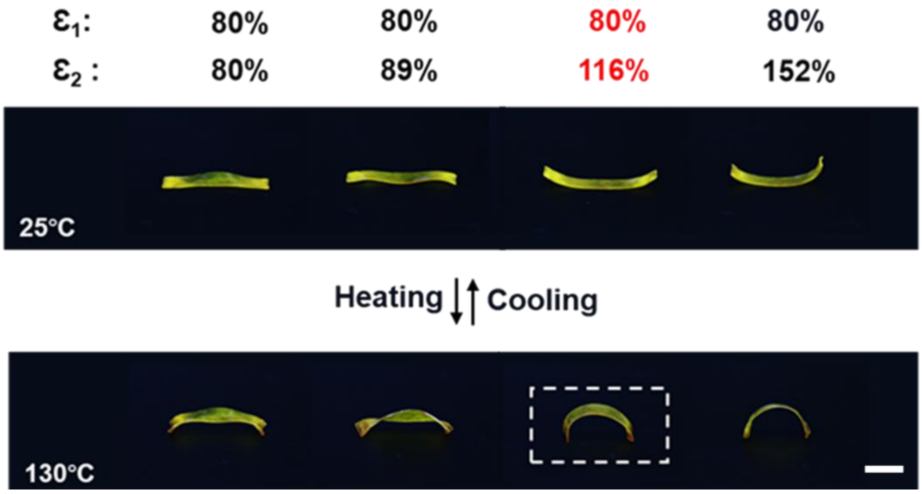

Figure S5. The heating and cooling states of the LCE samples obtained by varying the pre-stretch strain (ε_2) in the second stage of the mechanical training. The overall sample dimensions are fixed (length: 40 mm, width: 5 mm, thickness: 0.36 mm) Note: although the sample corresponding to ε_2 of 152% produces the largest actuation, its resistance to flip after collapse in the arched state (see Figure 2a) at 130 °C is too high. As such, it does not support the continuous movement behavior shown in Figure 2a.

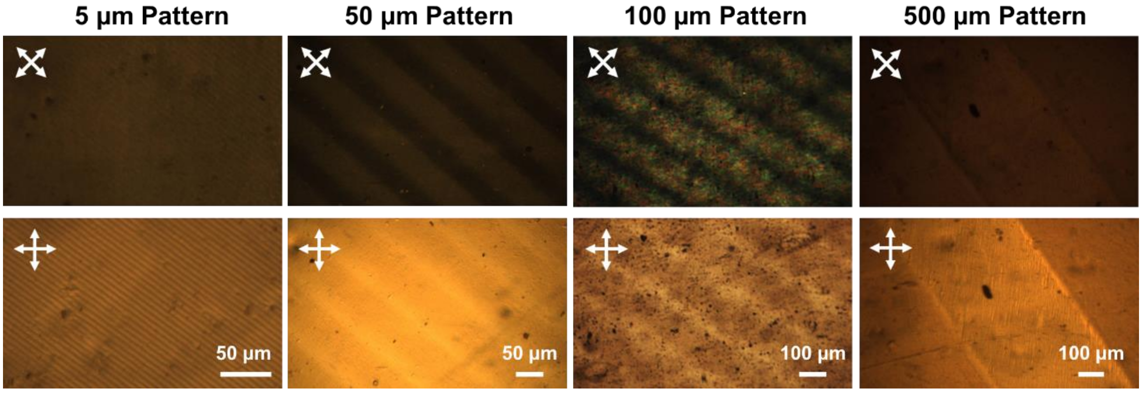

Figure. S6. The polarized optical micrographs of patterned LCE films corresponding to different pattern widths**.** It can be seen that boundaries with the enhanced orientation are clearly distinguishable based on the 500 μm pattern. This phenomenon can be primarily attributed to the intensified shear force resulting from different internal stresses in two regions, which arise due to their distinct moduli during stretching.

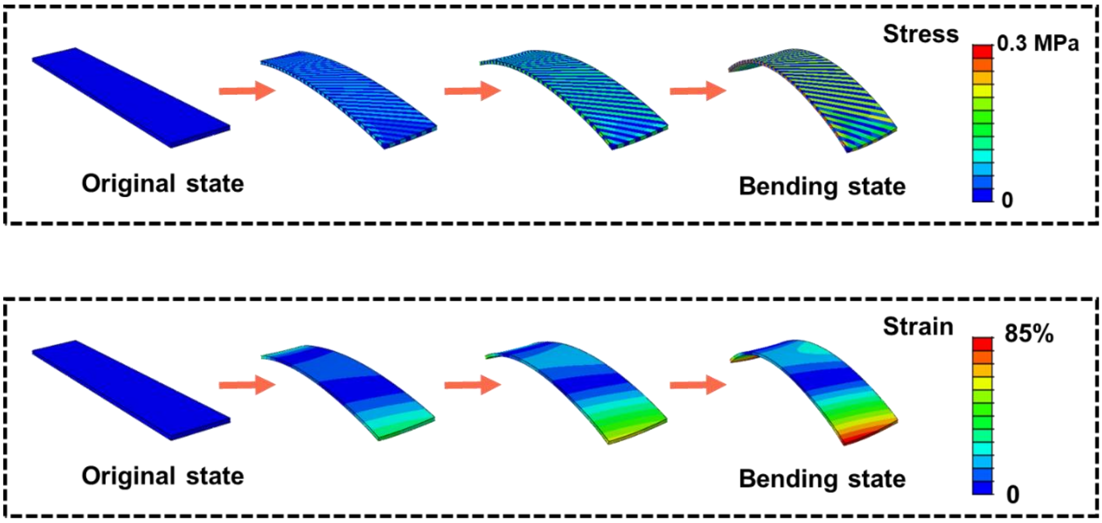

Figure S7. Finite element simulation of the shape-morphing behavior of the LCE film on the hot plate after mechanical training.

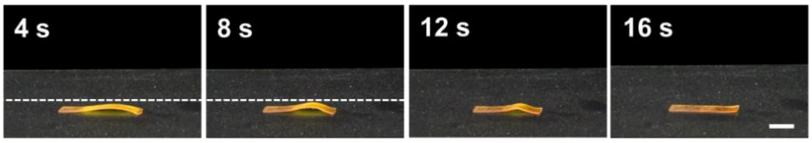

Figure S8. The behavior of non-patterned LCE materials on the 120 °C hot plate (scale bar: 1 cm). After being placed on the hotplate, the bottom side undergoes heating and contracts before the upper side, leading to up-arching. With further heating, the LCE eventually attains a uniform high temperature (above T_{NI}) and is in the uniformly contracted state. No flipping is observed in the process.

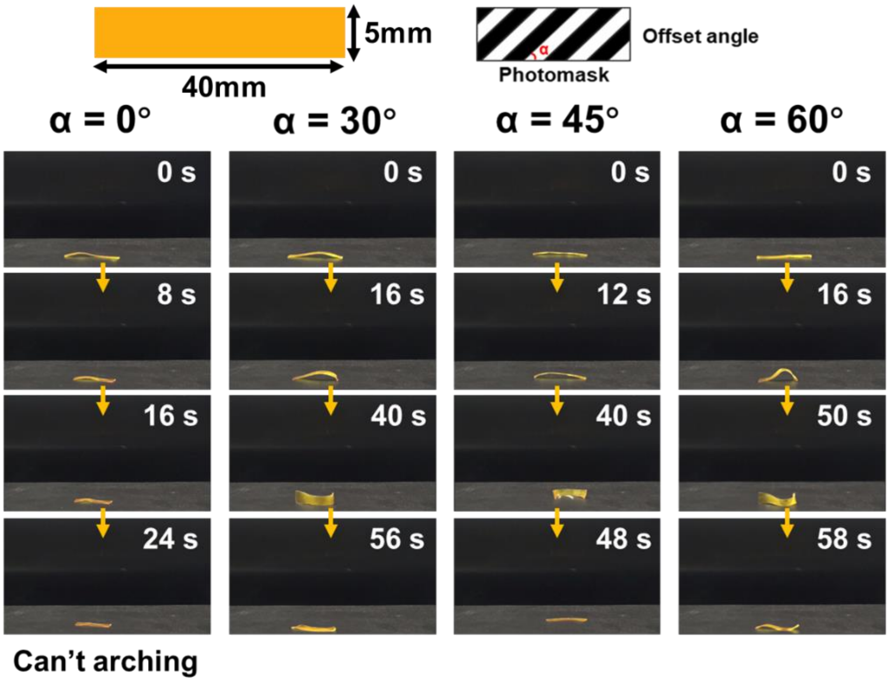

Figure S9. The heating induced (130 °C) actuation of the LCE samples fabricated by varying the offset angle. For the offset angle of 0, the sample contracts but does not arch. The arching is similar for the LCEs corresponding to offset angles of 30, 45, and 60 degrees.

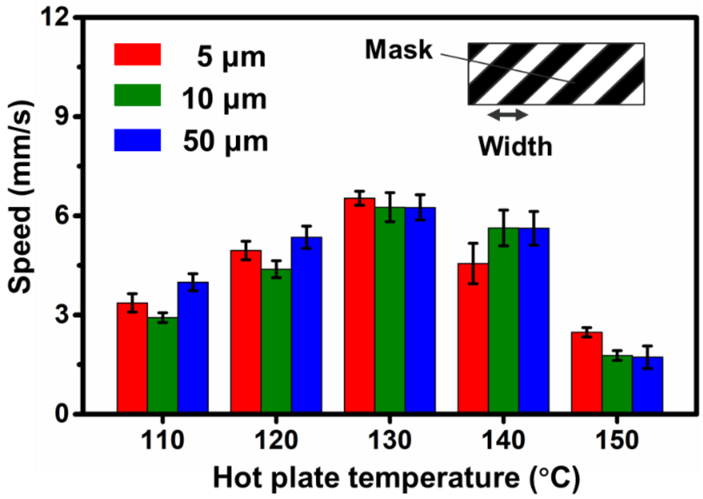

Figure S10. The dependence of the moving speed on the hot plate temperature for LCEs corresponding to different pattern strip widths. The overall sample dimensions are fixed (length: 40 mm, width: 0.75 mm, thickness: 0.36 mm). The data suggests that the hotplate temperature has a large impact whereas the pattern strip width has insignificant influence on the moving speed.

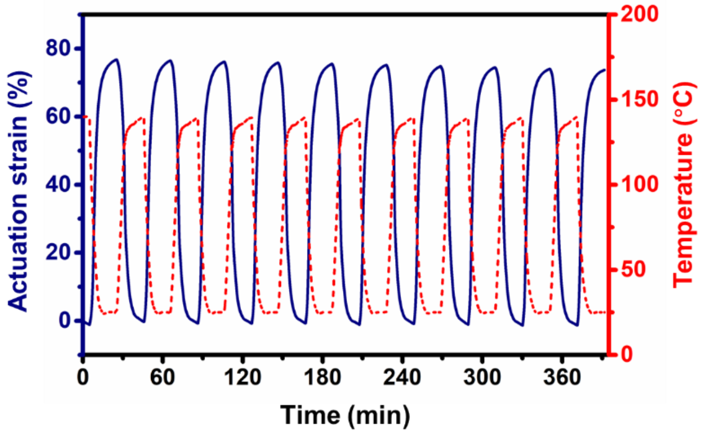

Figure S11. Cyclic actuation behavior of the LCE sample upon heating and cooling.

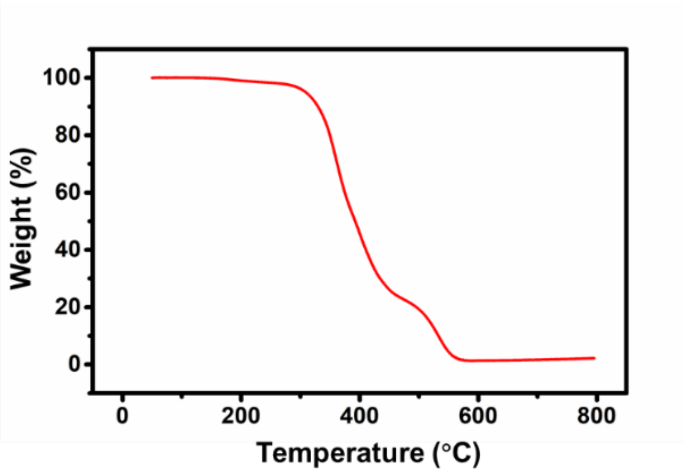

Figure S12. Thermogravimetric curve of the LCE (n_{PETMP}/n_{EDDET}=1/4).

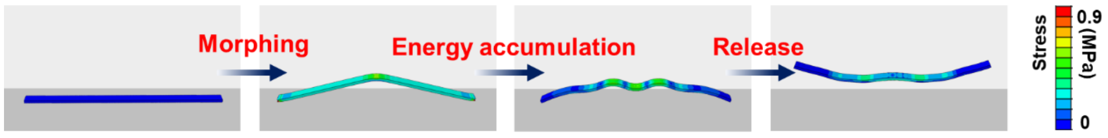

Figure S13. Finite element simulation of jumping process with a simplified model. A constraint was utilized to the model during the up-arching process. when the energy accumulates to a certain degree, its sudden release would lead to a jumping motion.

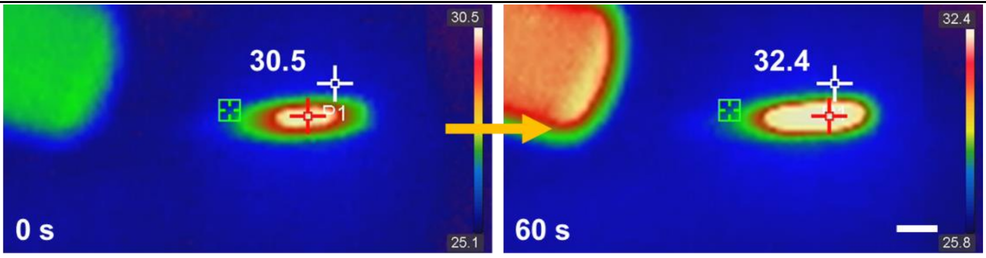

Figure S14. The thermal infrared imagery of photo-responsive LCE under UV illumination (365 nm, 90 mW/cm^2) for 60 s. The temperature increased by 1.9 degrees after 60s irradiation.

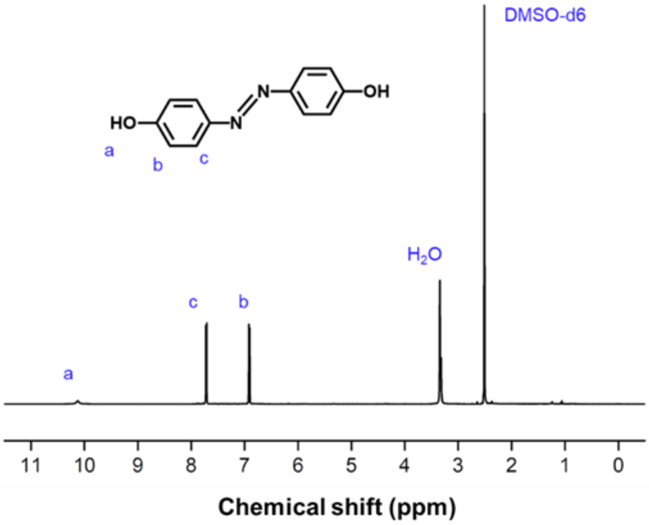

Figure S15. The ^{1}H NMR spectra of 4,4′-dihydroxyazobenzene in DMSO-d6.

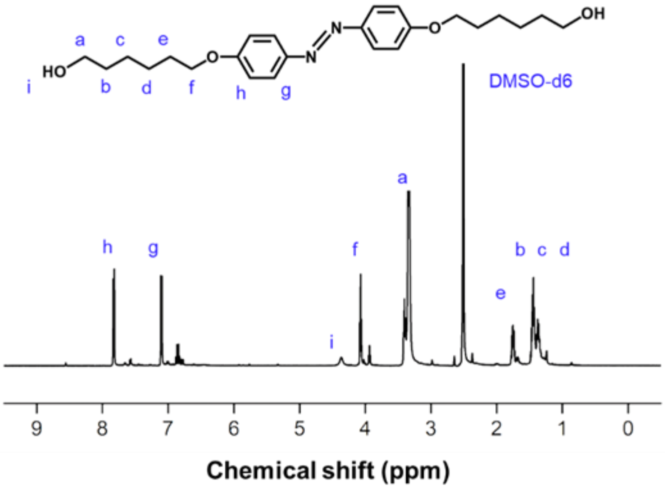

Figure S16. The ^{1}H NMR spectra of 4,4'-bis(6-hydroxyhexyloxy) azobenzene in DMSO-d6.

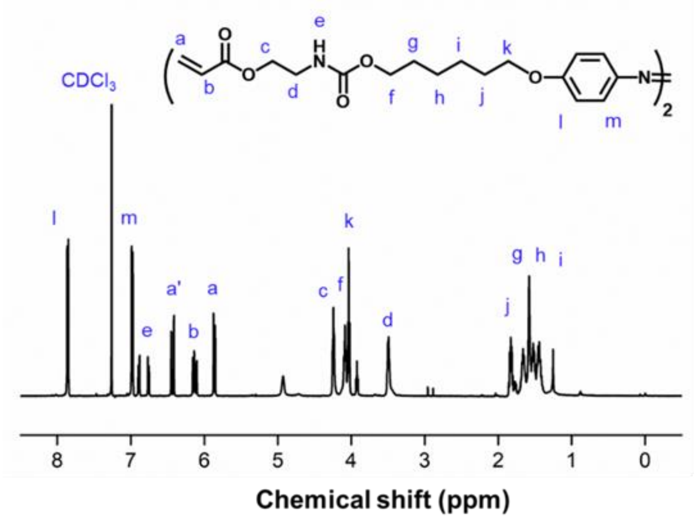

Figure S17. The ^{1}H NMR spectra of azobenzene containing acrylate in $CDCl_3$.

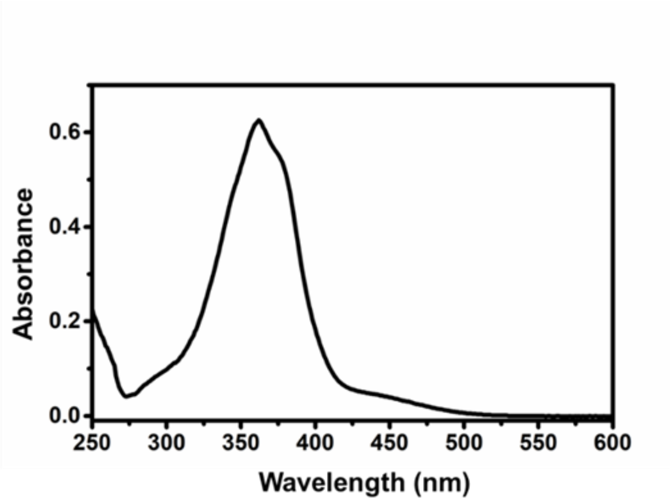

Figure S18. UV-Vis spectra of the azobenzene containing acrylate in solution (100 ppm in DMF)

Supplementary Movie S1: Self-sustained random movement.
Supplementary Movie S2: Self-sustained straight-line movement and circular movement.
Supplementary Movie S3: Autonomous jumping motion.
Supplementary Movie S4: The combined autonomous rolling and jumping.
Supplementary Movie S5: Light-gated movement.
Supplementary Movie S6: Drive around obstacles.
Supplementary Movie S7: Thermotaxis of LCE.
Supplementary Movie S8: Release trapped LCE.
Supplementary Movie S9: Self-sustained movement underwater.
